# Scale-up of an amoeba-based process for the production of the cannabinoid precursor olivetolic acid

**DOI:** 10.1186/s12934-022-01943-w

**Published:** 2022-10-20

**Authors:** Johann E. Kufs, Christin Reimer, Emily Steyer, Vito Valiante, Falk Hillmann, Lars Regestein

**Affiliations:** 1 Bio Pilot Plant, Leibniz Institute for Natural Product Research and Infection Biology – Hans Knöll Institute (Leibniz-HKI), Jena, Germany; 2 Evolution of Microbial Interactions, Leibniz Institute for Natural Product Research and Infection Biology – Hans Knöll Institute (Leibniz-HKI), Jena, Germany; 3 Faculty of Biological Sciences, Friedrich Schiller University Jena, Jena, Germany; 4 Biobricks of Microbial Natural Product Syntheses, Leibniz Institute for Natural Product Research and Infection Biology – Hans Knöll Institute (Leibniz-HKI), Jena, Germany; 5 Present Address: Biochemistry/Biotechnology, Faculty of Engineering, Hochschule Wismar University of Applied Sciences Technology, Business and Design, Wismar, Germany

**Keywords:** Amoeba, *Dictyostelium discoideum*, Cannabinoids, Local energy dissipation

## Abstract

**Background:**

The availability of new biological platform organisms to get access to innovative products and processes is fundamental for the progress in biotechnology and bioeconomy. The amoeba *Dictyostelium discoideum* represents a novel host system that has recently been employed for both the discovery of new natural products and as a cell factory for the production of bioactive compounds such as phytochemicals. However, an essential parameter to evaluate the potential of a new host system is the demonstration of its scalability to allow industrial applicability. Here, we aimed to develop a bioprocess for the production of olivetolic acid, the main precursor of cannabinoids synthesized by a recently engineered *D. discoideum* strain.

**Results:**

In this study, a sophisticated approach is described to scale-up an amoeba-based polyketide production process in stirred tank bioreactors. Due to the shear sensitivity of the cell wall lacking amoebae, the maximum local energy dissipation rate (*ε*_*max*_) was selected as a measure for the hydromechanical stress level among different scales. By performing 1.6-L scale batch fermentations with different stress conditions, we determined a maximum tolerable *ε*_*max*_ of 3.9 W/kg for *D. discoideum*. Further, we used this parameter as scale-up criterion to develop a bioprocess for olivetolic acid production starting from a 7-L stirred tank reactor to the industrially relevant 300-L scale with a product concentration of 4.8 µg/L, a productivity of 0.04 µg/L/h and a yield of 0.56 µg/g glucose.

**Conclusion:**

We developed a robust and reliable scale-up strategy for amoeba-based bioprocesses and evaluated its applicability for the production of the cannabinoid precursor olivetolic acid. By determining the maximum tolerable hydromechanical stress level for *D. discoideum*, we were able to scale-up the process from shake flasks to the 300-L stirred tank reactor without any yield reduction from cell shearing. Hence, we showed the scalability and biotechnological exploitation of amoeba-based processes that can provide a reasonable alternative to chemical syntheses or extractions of phytochemicals from plant biomass.

**Supplementary Information:**

The online version contains supplementary material available at 10.1186/s12934-022-01943-w.

## Background

Natural products present a major treasure trove for the discovery of new drug candidates with a wide range of biological activities [1–4]. Besides classical sources for natural products such as bacteria, yeast or plants, the amoeba *Dictyostelium discoideum* has a high potential not only for the discovery of new metabolites but also to serve as production platform for existing compounds. The presence of up to 45 polyketide synthase (PKS) genes within the genome of *D. discoideum* demonstrates the huge genetic prerequisite to produce polyketides and suggests a high availability of corresponding precursors [5]. Additionally, the amoeba can be genetically modified to generate complex phytochemicals with therapeutic relevance. We have recently shown that *D. discoideum* is amenable to synthesize a variety of polyketides after expression of the respective PKS genes [6–8]. Further, we previously engineered an amoeba/plant inter-kingdom hybrid enzyme that produced olivetolic acid (OA) as the key intermediate of the therapeutically valuable cannabinoids from the plant *Cannabis sativa*. This unique hybrid enzyme presents a novel biosynthetic approach for the production of cannabinoids [6]. Previously, common microbial hosts such as *Escherichia coli* or *Saccharomyces cerevisiae* have been used to synthesize OA or cannabinoids [9, 10]. However, none of them are considered to be suitable producers of polyketides, meaning that they have been genetically optimized for the production of relevant precursors. Especially the need for hexanoyl-CoA in the OA biosynthesis often requires the implementation of accessory pathways (Table 1). Our engineered amoeba harboring the hybrid PKS enzyme, however, already provides the hexanoyl-CoA moiety without any pathway optimizations or precursor supplementations. In addition, functional expression of tailoring enzymes from the cannabinoid biosynthetic pathway, *e.g.* the aromatic prenyltransferase and cyclizing oxidoreductases, requires post-translational modifications and membrane localization that are provided by amoebae but difficult to implement in prokaryotic hosts such as *E. coli* [11]. Although OA concentrations produced by the engineered *D. discoideum* strain were far below the range of milligrams per liter as shown for *S. cerevisiae* or *E. coli*, concentrations of native polyketides were reaching this range [6, 8], showing the potential for heterologous polyketide biosyntheses upon further optimization.

**Table 1:** Olivetolic acid production using different microbial hosts

Organism	PS	PO	TEE	Scalability	OA production	References
*E. coli*	+	+	–	+	80 mg/L	Tan, 2018 [10]
*S. cerevisiae*	+	–	+	+	0.48 mg/L	Gagne, 2012 [12]
*S. cerevisiae*	–	+	+	+	1.5 mg/L	Luo, 2019 [9]
*A. nidulans*	–	–	+	+/–	80 mg/L	Okorafor, 2021 [13]
*D. discoideum*	–	–	+	+	4.5 µg/L	Reimer, 2022 [6]

PS: precursor supplementation; PO: pathway optimization; TEE: tailoring enzyme expression; OA: olivetolic acid

An essential part of innovative bioprocesses and bioproducts is the demonstration of their scalability. In case of a newly discovered metabolic product, generating suitable amounts of pure compound is necessary to disclose the chemical structure and biological function. Moreover, scalability is one of the most important criteria to evaluate the potential for commercialization of a new product. In general, there are three geometrically independent scale-up strategies, namely the mixing time, gas transfer and volumetric power input. Since amoebae containing culture broth can be described as water-like solution without any elevated viscosity, the mixing time, recirculation and related aspects of flow behavior are not critical issues for scale-up. With respect to gas transfer, a sufficient oxygen supply is essential for an unlimited metabolic activity as amoeba cells are fully aerobic growing organisms [14]. Nevertheless, with a maximum oxygen uptake rate below 10 mmol/L/h, the oxygen transfer will not be a limiting factor even in industrial scale (> 100 m^3^). Inhibitions due to metabolically generated carbon dioxide in liquid phase are not known so far.

Without any cell wall and an average size of 11 µm, *D. discoideum* cells are consequently sensitive to hydromechanical forces [15]. Therefore, power input and related process parameters are critical for amoeba-based cultivations and scale-up. However, there are no processes for secondary metabolite production with *D. discoideum* existing so far, as this has only recently become biotechnologically relevant. Common cultivations with amoebae are performed in shake flasks [16, 17], presenting a suitable approach for shear-sensitive cells. There are only a few scale-up and transfer studies of amoebae from shake flask into lab-scale stirred tank reactors (STRs), that revealed a stirring rate of 150 rpm as a crucial parameter for the initial growth, and showed a maximum cell number of 5.5 × 10^7^ cells/mL [18–20]. Thus, sensitivity of the amoeba to hydromechanical stress can be assumed.

A common approach to evaluate the hydromechanical stress introduced by different stirrer types in one system is the stirrer tip speed, which however, does not take into account the scale and geometry of the bioreactor. Hence, a more effective way of evaluating the hydromechanical stress among different scales is the ratio between maximum local energy dissipation rate (*ε*_*max*_) to volume average energy dissipation rate (*ε*_*0*_) reported for unaerated systems [21, 22]. Since aeration is an essential parameter for the majority of biotechnological processes, Daub et al. developed and applied a method considering this effect on the hydromechanical stress [23, 24].

**Fig. 1:**
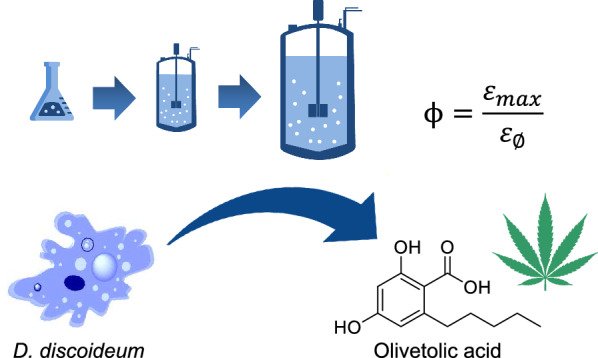
Scale-up strategy for olivetolic acid production with the amoeba *Dictyostelium discoideum*

In this study, we describe a sophisticated approach to scale-up an amoeba-based polyketide production process into a 300-L STR using the criterion of *ε*_*max*_ as a measure for the hydromechanical stress level among different scales (Fig. 1). Thereby, we demonstrated the suitability of *D. discoideum* as biotechnological host strain for the production of plant-derived pharmaceuticals in commercially relevant scales.

## Results

### The tolerable maximum local energy dissipation of *D. discoideum*

**Fig. 2:**
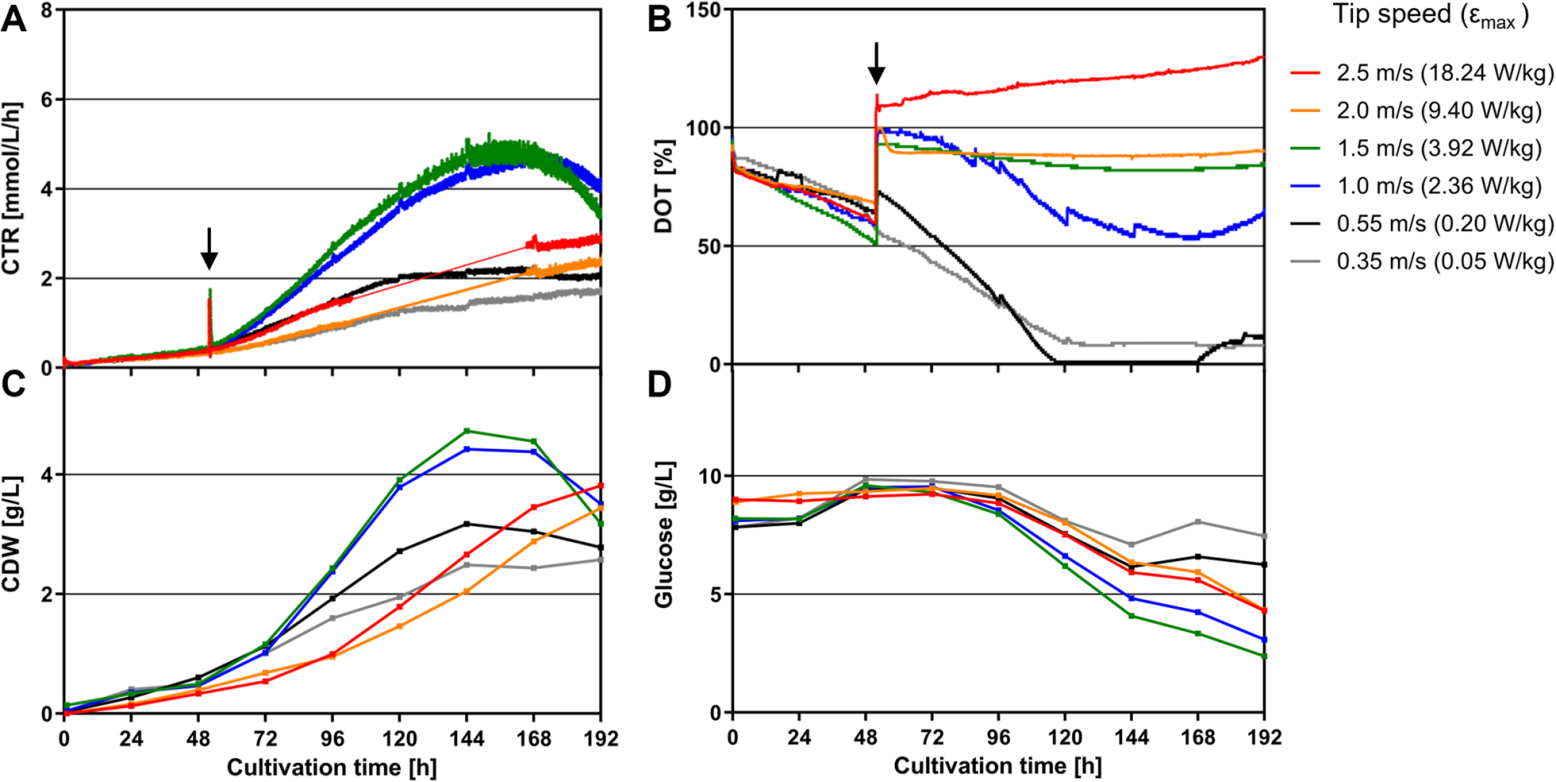
Influence of *ε*_*max*_ on growth of the *D. discoideum* pChR7 strain. During the lag phase, the stirrer tip speed was kept constant at 0.35 m/s for 52 h. After reaching the exponential growth phase (black arrow), the stirrer speed was changed as indicated. The growth behavior was monitored by measuring the carbon dioxide transfer rate (CTR) (**A**), dissolved oxygen tension (DOT) (**B**), the cell dry weight (CDW) (**C**) and glucose consumption (**D**) for the different stirrer tip speeds. Culture conditions: 1.6-L STR; HL5 medium with 10 g/L glucose, 20 µg/mL G418 and 20 µg/mL doxycycline; aeration rate: 0.3 vvm; temperature 22 °C; pH = 6.5 ± 0.05

**Fig. 3:**
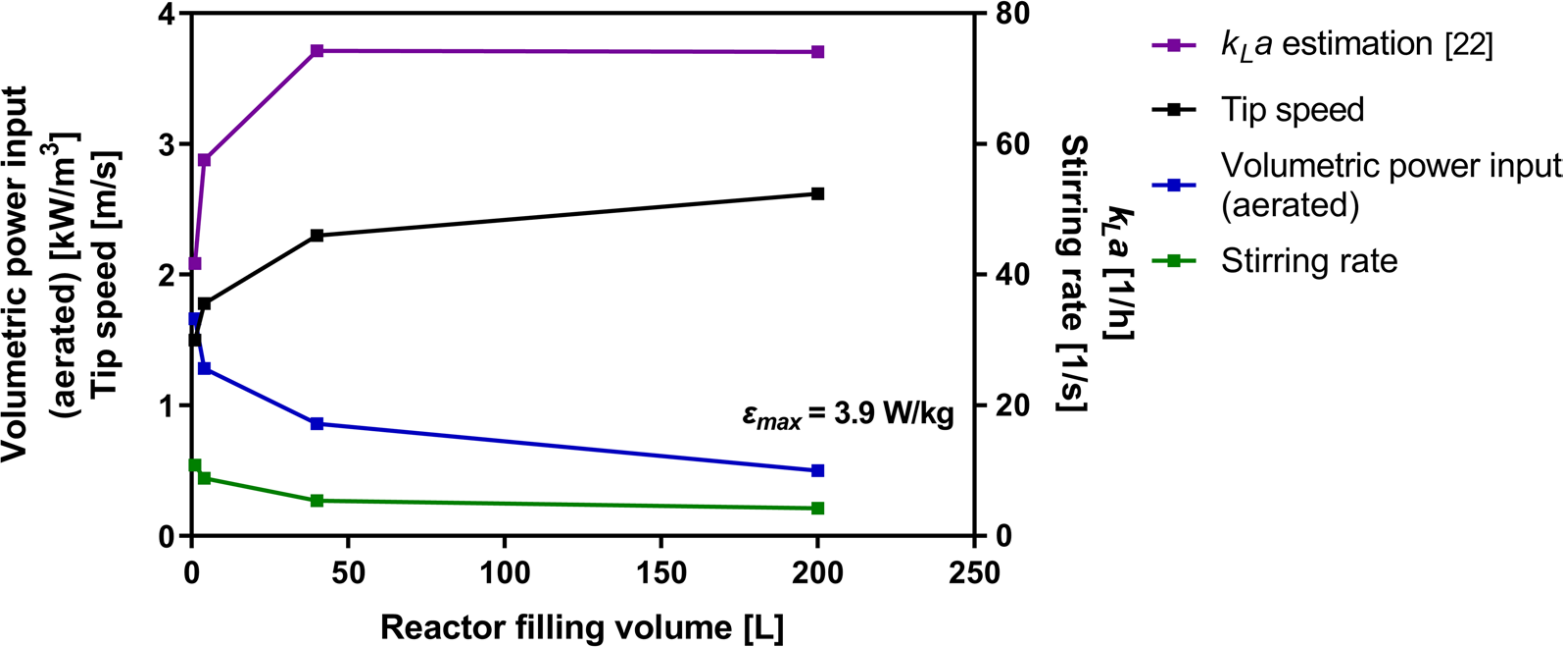
Influence of constant *ε*_*max*_ on the fermentation parameters in larger reactor systems. At constant *ε*_*max*_, an increase in tip speed and *k*_*L*_*a* can be observed, while the stirring rate and volumetric power input are decreasing with increasing reactor filling volume. All necessary geometric details and calculated values for the 1.6-L, 7-L, 75-L and 300-L reactor systems are presented in Table 2

A key aspect for the cultivation of *D. discoideum* in STRs is the predominant hydromechanical stress in the system introduced by the stirring rate [20]. We determined the maximum tolerable hydromechanical stress level of *D. discoideum* by performing six 1.6-L scale batch fermentations with different stress conditions (Fig. 2). First, the amoebae were allowed to adapt from shake flasks to the altered conditions in the bioreactor at a low stirring rate with a tip speed of 0.35 m/s as reported in the literature for a lag phase of approximately 50 h [18]. After the lag phase, a stirrer profile was applied to the indicated tip speeds within 6 min to achieve different stirring rates. It can be clearly seen that at stirrer tip speeds of 0.35 and 0.55 m/s, an oxygen limitation occurred as demonstrated by a decreasing dissolved oxygen tension (DOT), a plateau of the carbon dioxide transfer rate (CTR) curve and a reduced biomass formation (Fig. 2A, C). The slow cell growth is also reflected by a relatively low maximum specific growth rate (*µ*_*max(CTR)*_) of 0.019 h^−1^ for both conditions. Higher stirring rates of 1 and 1.5 m/s led to normal cell growth with *µ*_*max(CTR)*_ of 0.026 h^−1^ and 0.028 h^−1^, respectively. Notably, no oxygen limitation can be observed for those conditions (Fig. 2B). Further increase of the stirring rate to 2 and 2.5 m/s under fully oxygen saturated conditions, however, resulted in a decreased growth with *µ*_*max(CTR)*_ of 0.018 h^−1^ and 0.02 h^−1^, respectively. The gradation of the glucose consumption goes along with the detected growth profiles (Fig. 2D). The unlimited growth of amoebae at a stirrer tip speed of 1.5 m/s suggests this condition as the upper limit of the maximum tolerable hydromechanical stress for *D. discoideum.* Based on Eqs. 1–6 and geometric parameters (Table 2), a maximum local energy dissipation rate (*ε*_*max*_) of 3.9 W/kg was calculated and used as criterion for further scale-up experiments. As a consequence of constant *ε*_*max*_, the stirring rate and volumetric power input under aerated conditions decrease with an increasing reactor filling volume (Fig. 3). An opposite effect is visible for the tip speed which increases from 1.5 m/s to 2.6 m/s. The oxygen supply, indirect represented by the estimated volumetric gas transfer coefficient *k*_*L*_*a*, is slightly increasing from 1.6-L to 75-L scale [25]. Therefore, a negative influence of oxygen limitation on the scale-up of the process can be excluded.

**Table 2:** Geometrical details of reactors and parameters used for scale-up experiments

Parameter	Symbol	1.6-L STR	7-L STR	75-L STR	300-L STR
Reactor configurations					
Filling volume [m^3^]	${V}_{L}$	0.001	0.004	0.04	0.2
Reactor diameter [m]	${D}_{R}$	0.11	0.16	0.3	0.6
Stirrer diameter [m]	${d}_{r}$	0.044	0.064	0.135	0.196
Stirrer height [m]	${h}_{r}$	0.009	0.015	0.027	0.04
Number of stirrers	${S}_{N}$	2	2	2	2
Scale-up parameters (Eqs. 1–6)					
Maximum local energy dissipation rate [W/kg]	${\varepsilon }_{max}$	3.92	3.92	3.92	3.92
Ratio of maximum to specific energy dissipation rate (aerated)	${\Phi }_{g}$	2.36	3.02	4.58	7.83
Ratio of maximum to specific energy dissipation rate (unaerated)	$\Phi$	36.54	41.45	51.75	82.86
Estimated volumetric gas transfer coefficient [1/h] [25]	${k}_{L}a$	41.7	57.6	74.3	74.1
Volumetric power input (aerated) [kW/m^3^]	${P}_{g}$	1.66	1.3	0.86	0.50
Gas velocity [m/s]	${u}_{g}$	5.26 × 10^–4^	9.95 × 10^–4^	2.83 × 10^–3^	3.54 × 10^–3^
Power number	${P}_{O}$	8.26	7.6	6.5	5.56
Stirring rate [1/s]	${n}_{r}$	10.83	8.88	5.41	4.29

Power numbers were calculated according to Henzler et al. [26]

### Transfer of the OA production process from shake flask to 7-L bioreactor

**Fig. 4:**
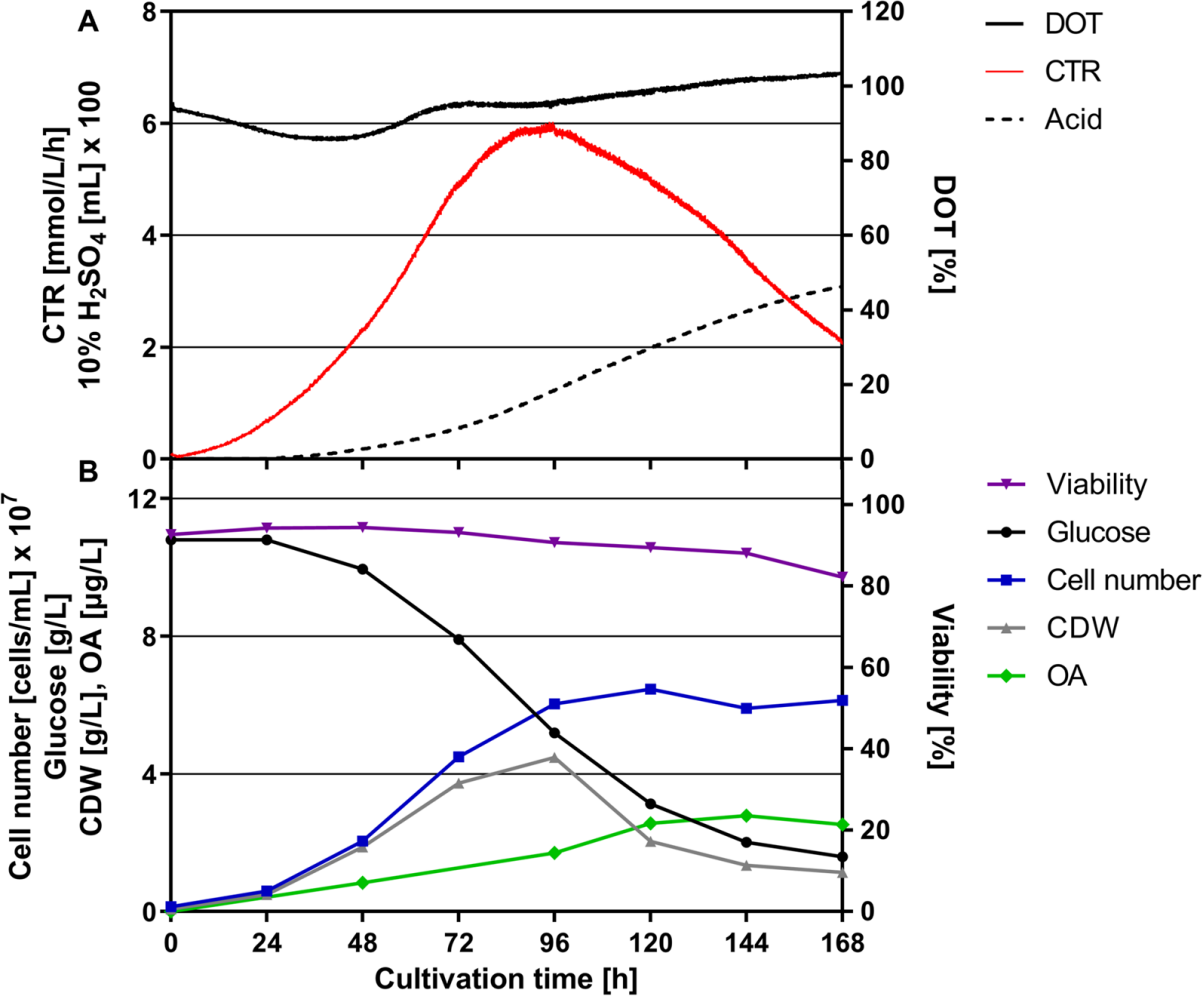
Scale-up of the OA production process from shake flask to 7-L STR based on *ε*_*max*_. Upon pre-culture in a 1.6-L STR with an adaption phase of 8 h and an *ε*_*max*_ of 3.9 W/kg, cells were transferred into a 7-L STR at a cell density of 1 × 10^7^ cells/mL. **A** Online parameters of *D. discoideum* pChR7 batch fermentation including carbon dioxide transfer rate (CTR, red), dissolved oxygen tension (DOT, black) and sulfuric acid consumption (dotted) were monitored. **B** Offline parameters such as cell dry weight (CDW, grey), cell number (blue), glucose consumption (black), viability (violet) and olivetolic acid production (OA, green) are depicted over the course of the fermentation. Culture conditions: 7-L STR; HL5 medium with 10 g/L glucose, 20 µg/mL G418 and 20 µg/mL doxycycline; pH = 6.5 ± 0.05 was maintained by the addition of 10% H_2_SO_4_; tip speed: 1.78 m/s; stirring rate = 530 rpm; aeration rate: 0.3 vvm; temperature 22 °C

As we were able to show that the hydromechanical stress has a crucial effect on growth of *D. discoideum*, we aimed to constantly keep the *ε*_*max*_ at 3.9 W/kg to ensure fully aerated and oxygen unlimited conditions at all time. In order to adapt the amoeba cells to this stress level, the pre-culture was grown for 48 h in a 1.6-L STR with a stirring rate switch after 40 h. For cultivation of amoebae in a 7-L bioreactor, the stirring rate of 530 rpm was calculated based on *ε*_*max*_ and used throughout the entire process as the cells are already adapted to the hydromechanical stress by the pre-culture set-up. After a short lag phase of approximately 24 h, the CTR increased exponentially and reached the maximum of 5.9 mmol/L/h after 94 h (Fig. 4A), while a maximum cell dry weight (CDW) of approximately 4.5 g/L and a glucose consumption of 8.4 g/L were obtained (Fig. 4B). This demonstrates that the lag phase can be significantly reduced by an optimized pre-culture regime, which is especially relevant for the slow growing amoeba cells. Additionally, a maximum cell concentration of 6.03 × 10^7^ cells/mL, a *µ*_*max(CTR)*_ of 0.036 h^−1^ and a constant cell viability over 90% reflect that the amoebae were able to cope with the hydromechanical stress in the STR without showing any characteristics of growth inhibition (Fig. 4B). Notably, the selected conditions were favorable for sufficient oxygen supply as the DOT remained above 85%, and resulted in an OA production of 2.53 µg/L with a productivity of 0.018 µg/L/h and a yield of 0.3 µg/g glucose (Fig. 4B).

### Scale-up to 300-L bioreactor for OA production

As scale-up criterion, the maximum local energy dissipation rate *ε*_*max*_ was selected due to the previously described sensitivity of the amoeba against shear forces. The conventional scale-up criteria such as the mass transfer coefficient *k*_*L*_*a* or volumetric power input are neglected since those parameters do not include the reactor geometry or are not suitable as oxygen supply is not a crucial parameter for the cultivation of *D. discoideum*. The advantage of applying *ε*_*max*_ is the simultaneous consideration of the power input, aeration and reactor geometry for the calculation of the hydromechanical stress.

**Fig. 5:**
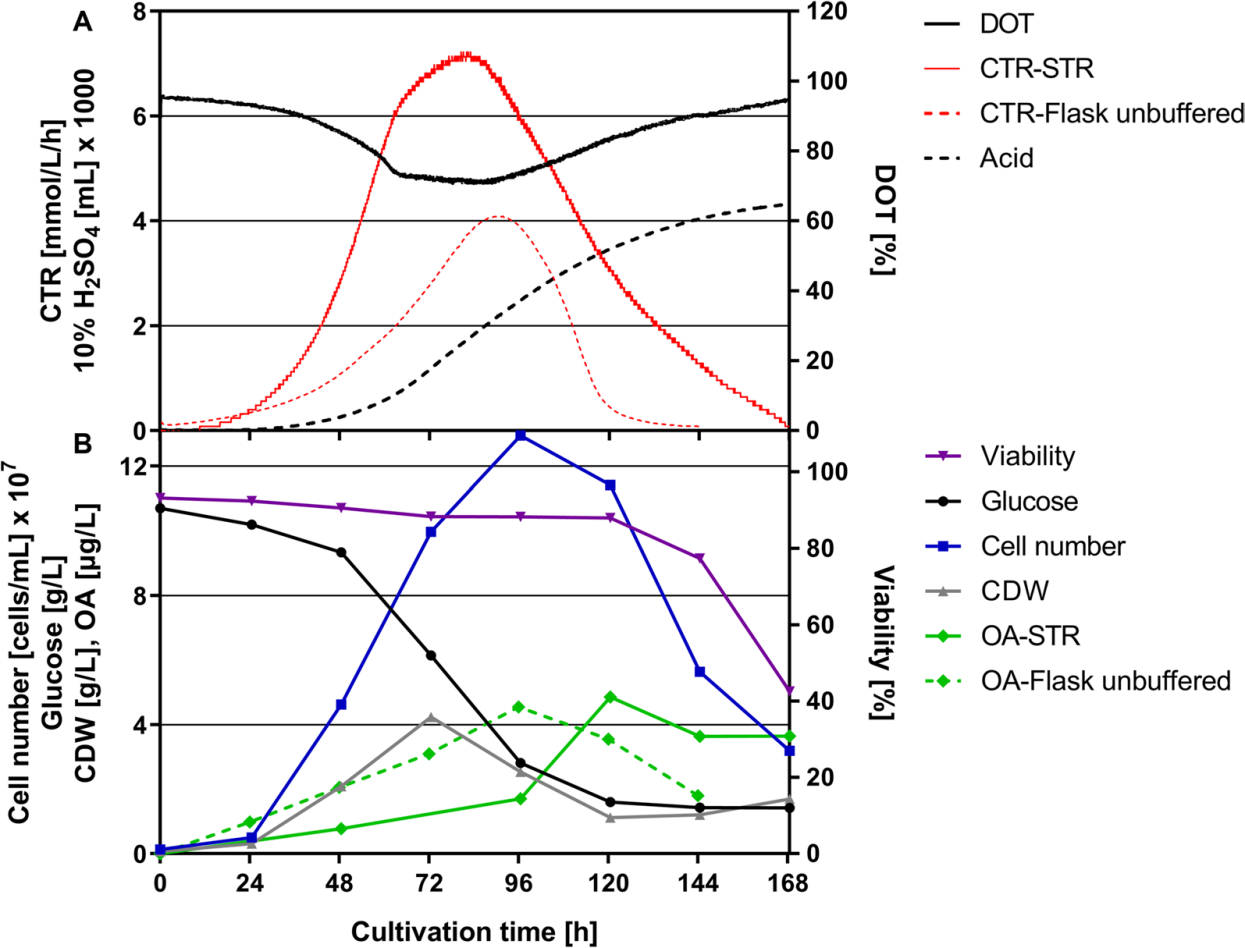
Scale-up of the OA production process from shake flask to 300-L STR based on *ε*_*max*_. After expanding the pre-culture regime from 1.6-L over 7-L to 75-L STR, cells were transferred into a 300-L STR at a cell density of 1 × 10^7^ cells/mL. **A** Online parameters of *D. discoideum* pChR7 batch fermentation including carbon dioxide transfer rate (CTR-STR, red solid; CTR-shake flask as reported in Reimer et al*.*, 2022, red dotted), dissolved oxygen tension (DOT, black solid) and sulfuric acid consumption (black dotted) were monitored. **B** Offline parameters such as cell dry weight (CDW, grey), cell number (blue), glucose consumption (black), viability (violet) and olivetolic acid production (OA-STR, green solid; OA-shake flask, green dotted) are depicted over the course of the fermentation. Culture conditions: 300-L STR; HL5 medium with 10 g/L glucose, 20 µg/mL G418 and 20 µg/mL doxycycline; pH = 6.5 ± 0.05 was maintained by the addition of 10% H_2_SO_4_; tip speed: 2.62 m/s; stirring rate = 255 rpm; aeration rate: 0.3 vvm; temperature 22 °C

In order to show that the amoeba-based process can also be applied to commercially relevant scales, we transferred the developed process to a 300-L STR with a filling volume of 200 L. Therefore, three pre-cultures were performed in 1.6-L, 7-L and 75-L scale bioreactors at constant *ε*_*max*_ of 3.9 W/kg to finally inoculate the main culture in the 300-L bioreactor. This pilot scale fermentation showed slightly better growth kinetics in comparison to the 7-L STR in terms of a maximum CTR of 7.2 mmol/L/h after 82 h (Fig. 5A), a maximum CDW of 4.2 g/L and a glucose consumption of 8.6 g/L (Fig. 5B). Interestingly, a maximum cell number of 1.2 × 10^8^ cells/mL was reached after 96 h, while a DOT above 70% was maintained over the course of the fermentation. The OA production peaked at 4.8 µg/L after 120 h (Fig. 5) with a productivity of 0.04 µg/L/h and a yield of 0.56 µg/g glucose. At that point, the cell viability dropped from 88 to 42% over the next 48 h. Besides similar CDWs in both STRs, the higher maximum cell number in the 300-L scale suggests a decrease of the average cell size (Additional file 1: Fig. S1). However, a significantly increased *µ*_*max(CTR)*_ of 0.065 h^−1^ shows that the hydromechanical stress conditions are tolerable and not growth-limiting for the amoebae.

## Discussion

Bioprocesses based on microbial host organisms provide an important alternative to the extraction of plant-derived active compounds from their native plant sources or chemical syntheses that are often commercially infeasible and require the usage of toxic chemicals [27, 28]. Microbial cell factories enable the reliable, reproducible and selective production of individual plant secondary metabolites by harnessing the genetic blueprints of the plants. We recently showed that the amoeba *D. discoideum* can be employed as chassis organism for the production of plant-derived polyketides, such as resveratrol or olivetolic acid, the key intermediate in the cannabinoid biosynthetic pathway [6]. To evaluate the application and commercialization potential of the amoeba, the scalability of novel bioprocesses with this organism will be a crucial criterion.

In this study, we developed a scale-up strategy for an amoeba-based process to produce olivetolic acid using the recently engineered amoeba strain. To date, there is only a limited number of studies reported in which amoebae have been successfully transferred from shake flasks into lab-scale stirred-tank bioreactors (STRs). As amoebae lack a protective cell wall and are sensitive to shear forces, a low stirring rate was shown to be a crucial parameter for the initial growth in STRs [18, 20].

To exploit the maximum tolerable stress level of *D. discoideum*, we first performed six 1.6-L scale fermentations with varying stirrer tip speeds after the initial growth phase. Our data revealed an unlimited growth of amoebae at a tip speed of 1.5 m/s indicating a proper balance between shear forces and oxygen supply. This is in line with the literature reporting a maximum tip speed of 2.2 m/s at the end of a STR batch fermentation [18]. However, the stirrer tip speed does not take into account the reactor geometry, scale and influence of aeration [23]. Therefore, we used the maximum local energy dissipation rate (*ε*_*max*_) as scale-up criterion to better evaluate the hydromechanical stress conditions among different scales*.* Based on the correlation (Eq. 2) described by Daub et al*.* [23], the tip speed of 1.5 m/s was converted to *ε*_*max*_ of 3.9 W/kg, which was kept constant for all scale-up experiments. This condition resulted in a sufficient oxygen supply in both the 7-L and 300-L scale STR as the DOT remained above 85% and 70%, respectively. Notably, the newly integrated adaptation phase of amoebae to the elevated hydromechanical stress level in the 1.6-L pre-culture STR led to a shortened lag phase of 24 h in larger scales and a significantly reduced overall process time. For comparison, lag phases of 48 to 72 h have been previously reported in the literature [18]. The successful process scale-up from 7-L to the 300-L scale was demonstrated by similar CDWs of approximately 4 g/L, a glucose consumption of 8 g/L and maximum CTR values between 6 and 7 mmol/L/h. Interestingly, a maximum cell number of 1.2 × 10^8^ cells/mL was reached using the 300-L STR, which is far above the cell densities described for shake flasks and STRs with 2–3 × 10^7^ and 5 × 10^7^ cells/mL, respectively [18–20]. A glucose consumption of 80% observed in all STRs goes along with experiments reported in the literature [17]. Usually, the remaining C-source indicates a limitation of a secondary substrate e.g. phosphate, nitrogen or trace elements. However, the limitation of cell growth is probably an effect of a pre-starvation factor aiding the amoebae to sense their density and start the expression of genes for cell aggregation at a given cell number [29, 30]. That hypothesis would also be an explanation why Fed-Batch cultivations do not lead to higher cell numbers [18]. When comparing the CTR profile of the 7-L and 300-L STRs with the previously reported shake flask experiments [6], it becomes obvious that the pH control in STRs has a huge positive impact on growth rate and maximum metabolic activity as demonstrated by the higher CTR_max_ values. Concerning the OA production, a similar profile can be observed for both the 7-L and 300-L STR with most of it being produced after reaching the highest cell concentration and CTR_max_. In the 300-L STR, a concentration of 4.8 µg/L was achieved which is twice as high as the amount reached in the 7-L scale. The discrepancy between both scales can be explained by the overall error for ${\Phi }_{g}$ (Eq. 2) of approximately 30% reported by Daub et al*.* [23]. This deviation consequently results in a range of 2.73–5.07 for the calculated *ε*_*max*_ value and, in turn, leads to a stirring rate of 472–580 rpm for the 7-L scale. Nevertheless, the amount reached in the 300-L STR is in good agreement with the OA concentration obtained in shake flasks [6] demonstrating the feasibility of the developed scale-up strategy for amoeba-based processes. This approach opens up the possibility to produce pharmaceutically valuable substances, such as cannabinoids in the novel amoeba host system on commercially relevant scales and provides a great potential for natural product research.

## Conclusions

In this study, we developed a scale-up strategy for an amoeba-based bioprocess and evaluated its applicability for the production of olivetolic acid (OA) as the main precursor of the cannabinoid biosynthetic pathway. Since amoeba cells are sensitive to hydromechanical stress, we determined the maximum tolerable local energy dissipation rate of 3.9 W/kg as scale-up criterion. By adapting the amoeba to this stress condition during the pre-culture, we were able to shorten the lag phase of amoeba-based processes and produced OA in the commercially relevant 300-L scale with similar yields as previously reported. Our scale-up approach enables the large-scale production of industrially relevant substances with amoebae, and may facilitate the discovery and functional characterization of novel natural products. Thereby, we demonstrated the feasibility of *D. discoideum* to be used as novel microbial host system for innovative biotechnological processes.

## Methods

### Amoeba strain

To scale-up the olivetolic acid (OA) generating bioprocess, the recently reported *D. discoideum* pChR7 strain expressing a novel biosynthetic pathway for OA production was used [6].

### Cultivation

*Dictyostelium discoideum* AX2 cells were grown in HL5 complex medium (Formedium) supplemented with 10 g/L glucose at 22 °C in petri dishes, shake flasks at 140 rpm or stirred tank bioreactors (STRs) at indicated stirrer speeds. Selection of recombinant amoebae was performed by adding 20 µg/mL G418 (InvivoGen). To induce the expression of the OA biosynthetic genes, main cultures were supplied with 20 µg/mL doxycycline (AppliChem).

### Pre-cultures in shake flasks

In order to circumvent the necessity of cell number determination, we investigated if the metabolic activity can be used as a transfer criterion. The respiration activity was measured by using the Kuhner TOM (transfer-rate online measurement) system equipped with oxygen partial pressure sensors and infrared sensors to calculate the oxygen transfer rate (OTR) and the carbon dioxide transfer rate (CTR) [31]. *D. discoideum* was grown at an initial density of 1 × 10^6^ cells/mL in 1-L TOM flasks with a filling volume of 200 mL HL5 medium supplemented with 10 g/L glucose and 20 µg/mL G418. When the pre-cultures reached a cell concentration of 1 × 10^7^ cells/mL and a CTR of 1.3 mmol/L/h (Additional file 1: Fig. S2), 100 mL were used for inoculation of the 1.6-L bioreactor.

### Main cultures in stirred tank bioreactors (STRs)

All main cultures were inoculated with 10% filling volume to reach a starting concentration of 1 × 10^6^ cells/mL and performed as batch cultures in STRs (1.6-L and 7-L: Sartorius, 75-L: bbi-biotech, 300-L: Sartorius/Frings) with parameters indicated in Table 2. To ensure turbulent conditions, Reynolds numbers were always over 10,000. The pH was adjusted to 6.5 by addition of sulfuric acid, and antifoam was added if necessary. The batch cultures were grown at a constant temperature of 22 °C and an aeration rate of 0.3 vvm.

#### Small scale STR cultivation for evaluation of *ε*_*max*_

To determine the maximum tolerable hydromechanical stress level of the *D. discoideum* pChR7 strain, six 1.6-L scale batch fermentations were performed with different stress conditions by applying stirrer tip speeds of 0.35, 0.55, 1, 1.5, 2, and 2.5 m/s. After inoculation with a starting cell density of 1 × 10^6^ cells/mL, the stirrer tip speed was kept constant at 0.35 m/s for 52 h to allow the amoeba to adopt to the altered conditions in the STR. After the lag phase, a stirrer profile was applied to the afore mentioned tip speeds within 6 min. To define a suitable scale-up criterion based on the optimal growth profile, the tip speeds were used to calculate the maximum local energy dissipation rate (*ε*_*max*_) as described below.

#### STR cultivation for scale-up

The scale-up of the OA production process to 300-L was performed using three seed bioreactors (1.6-L, 7-L and 75-L) based on *ε*_*max*_. In the first 1.6-L scale, the stirrer tip speed was kept constant at 0.35 m/s for 40 h as the uninduced amoebae exhibit a faster growth rate. Then, a stirrer profile to the determined *ε*_*max*_ within 6 min followed and cultivation was continued for 8 h or until reaching final cell density. For the 7-L and 75-L seed bioreactors, *ε*_*max*_ was constant from the beginning (Table 2), since the amoebae were adopted to the hydromechanical stress conditions in the first seed bioreactor. The transfer of the seed culture to the next scale was performed at a cell density of 1 × 10^7^ cells/mL. For comparison, a small scale 7-L STR was carried out starting from a 1.6-L STR seed culture.

### Analytics

#### Online analytics

For all STR experiments, the dissolved oxygen tension (DOT), temperature and pH were measured online. For 7-L, 75-L and 300-L scales, the O_2_ and CO_2_ concentrations were measured using a Rosemount NGA 2000 off-gas analyzer (Emerson) with a paramagnetic sensor and an infrared analyzer. For the 1.6-L scale, the O_2_ and CO_2_ concentrations were measured using the BlueVary off-gas analyzer (BlueSens gas sensor). The oxygen transfer rate (OTR) carbon dioxide transfer rate and CTR were calculated, as previously reported [32]. The value of the respiratory quotient was always “1”, since OTR and CTR were equal. Due to higher signal quality, only trends of the CTR are depicted.

#### Offline analytics

During fermentation, samples were taken at regular time intervals in order to get offline data of the running process. At the indicated time points, the cell concentration and viability were determined at a size range of 7.6 to 17.6 µm using a CASY Cell Counter and the respective analyzer system (model TT, equipped with a 60 µm capillary, OLS Bio). The cell dry weight (CDW) was determined by centrifugation of 10 mL culture broth at 4 °C and 800 g for 5 min. While the supernatant was stored for further analysis, the cell pellet was washed with 5 mL of phosphate buffer (5 mM Na_2_HPO_4_, 5 mM KH_2_PO_4_, 1 mM CaCl_2_, 2 mM MgCl_2_), lyophilized overnight and weighed afterwards. For determination of the glucose concentration, 1 mL of the supernatant was measured using the YSI 2950 Analyzer (Kreienbaum). Moreover, 10 mL of culture broth were harvested for OA extraction and quantification using high performance liquid chromatography (HPLC) coupled with high resolution mass spectrometry (HRMS) as recently reported [6].

## Mathematical formulae

The maximum local energy dissipation rate can be calculated by the following equations (Eqs. 1–6).

1 $${\varepsilon }_{max}={\varPhi }_{g}\cdot {\varepsilon }_{\varnothing }$$

2 $${\varPhi }_{g}=2.3\cdot {\varPhi }^{0.34}\cdot {D}_{R}^{0.543}$$

3 $$\varPhi =\frac{4}{\pi }\cdot \frac{{V}_{L}}{{d}_{r}^{2}\cdot {h}_{r}}\cdot \frac{1}{{S}_{N}}$$

4 $${\varepsilon }_{\varnothing }=\frac{{P}_{g}}{\rho\cdot {V}_{L}}$$

5 $${P}_{g}= {P}_{0}\cdot \left[{{\left(1.384+{\left(735\cdot {\frac{{u}_{g}}{\sqrt{g\cdot {D}_{R}}}}\right)^{2}}\right)}}^{-0.5}+0.15\right]$$

6 $${P}_{0}=Po\cdot \rho \cdot {n}_{r}^{3}\cdot {d}_{r}^{5}$$

## Supplementary Information

**Additional file 1: Fig. S1.** Correlation of the cell number and CDW for the 1.6-L, 7-L and 300-L STR. A higher cell number per generated biomass of *D. discoideum* is shown for the 300-L scale suggesting a decrease in overall cell size. **Fig. S2.** Pre-culture set-up of pChR7 strain via transfer rate online measurement (TOM). For the pre-culture, a 1-L TOM flask with a filling volume of 250 mL was inoculated at 1 × 10^6^ cells/mL from a 50 mL liquid culture. After reaching a cell density of 1 × 10^7^ cells/mL, which corresponded to a CTR of 1.3 mmol/L/h, cells were used to inoculate the 1.6-L STR. Culture conditions: HL5 medium with 10 g/L glucose, 20 µg/mL G418; agitation: 140 rpm; temperature: 22 °C.

## Data Availability

The datasets used and/or analysed during the current study are available from the corresponding author on reasonable request.
