# Supplementary material for: Scale-up of an amoeba-based process for the production of the cannabinoid precursor olivetolic acid
Source: Microb Cell Fact. 2022 Oct 20;21:217. doi: 10.1186/s12934-022-01943-w (PMC9585784; doi:10.1186/s12934-022-01943-w)
Supplement: Supplementary file 1 — Additional file 1: Fig. S1. Correlation of the cell number and CDW for the 1.6-L, 7-L and 300-L STR. A higher cell number per generated biomass of D. discoideum is shown for the 300-L scale suggesting a decrease in overall cell size. Fig. S2. Pre-culture set-up of pChR7 strain via transfer rate online measurement (TOM). For the pre-culture, a 1-L TOM flask with a filling volume of 250 mL was inoculated at 1 × 10^6^ cells/mL from a 50 mL liquid culture. After reaching a cell density of 1 × 10^7^ cells/mL, which corresponded to a CTR of 1.3 mmol/L/h, cells were used to inoculate the 1.6-L STR. Culture conditions: HL5 medium with 10 g/L glucose, 20 µg/mL G418; agitation: 140 rpm; temperature: 22 °C. [file 12934_2022_1943_MOESM1_ESM.docx]

**Supplementary Information**

**for**

**Scale-up of an amoeba-based process for the production of the cannabinoid precursor olivetolic acid**

Johann E. Kufs^1^, Christin Reimer^2,3^, Emily Steyer^1^, Vito Valiante^4^, Falk Hillmann^2,5^ and Lars Regestein^1,#^

^1^Bio Pilot Plant, Leibniz Institute for Natural Product Research and Infection Biology – Hans Knöll Institute (Leibniz-HKI), Jena, Germany

^2^Evolution of Microbial Interactions, Leibniz Institute for Natural Product Research and Infection Biology – Hans Knöll Institute (Leibniz-HKI), Jena, Germany

^3^Faculty of Biological Sciences, Friedrich Schiller University Jena, Jena, Germany

^4^Biobricks of Microbial Natural Product Syntheses, Leibniz Institute for Natural Product Research and Infection Biology – Hans Knöll Institute (Leibniz-HKI), Jena, Germany

^5^present address: Biochemistry/Biotechnology, Faculty of Engineering, Hochschule Wismar University of Applied Sciences Technology, Business and Design, Wismar, Germany

^#^Corresponding author: lars.regestein@leibniz-hki.de


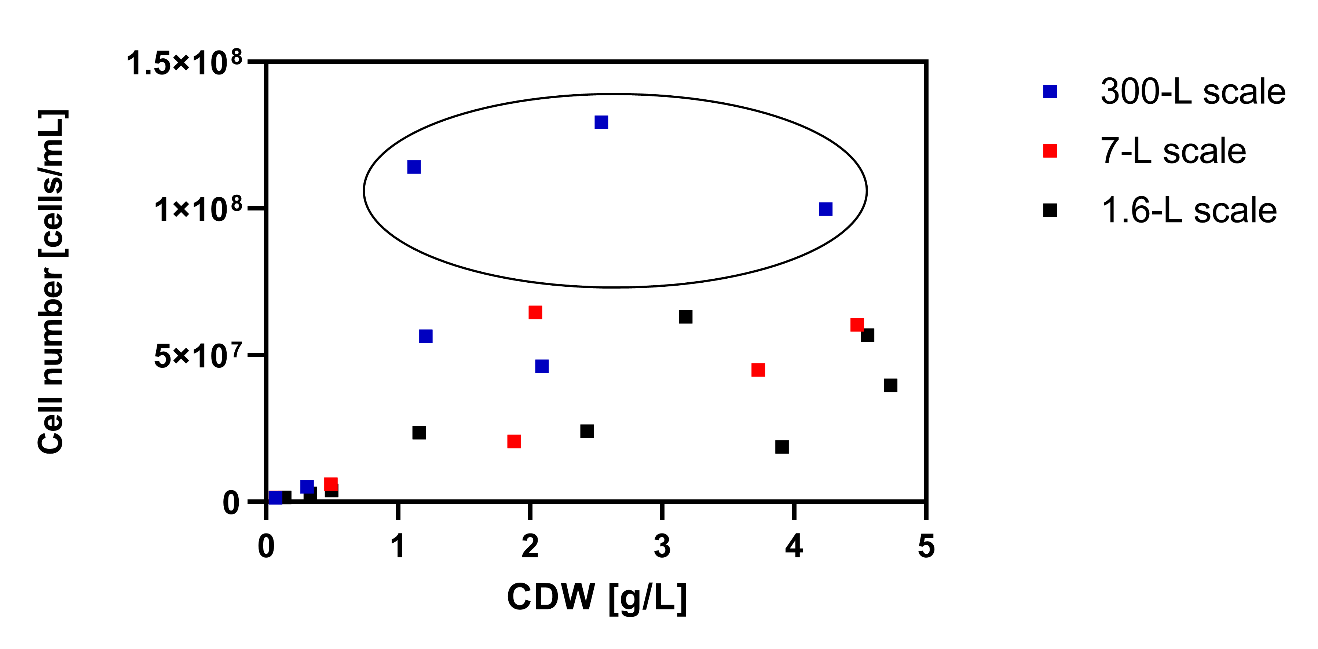


**Supplementary Fig. 1 | Correlation of the cell number and CDW for the 1.6-L, 7-L and 300-L STR.** A higher cell number per generated biomass of *D. discoideum* is shown for the 300-L scale suggesting a decrease in overall cell size.


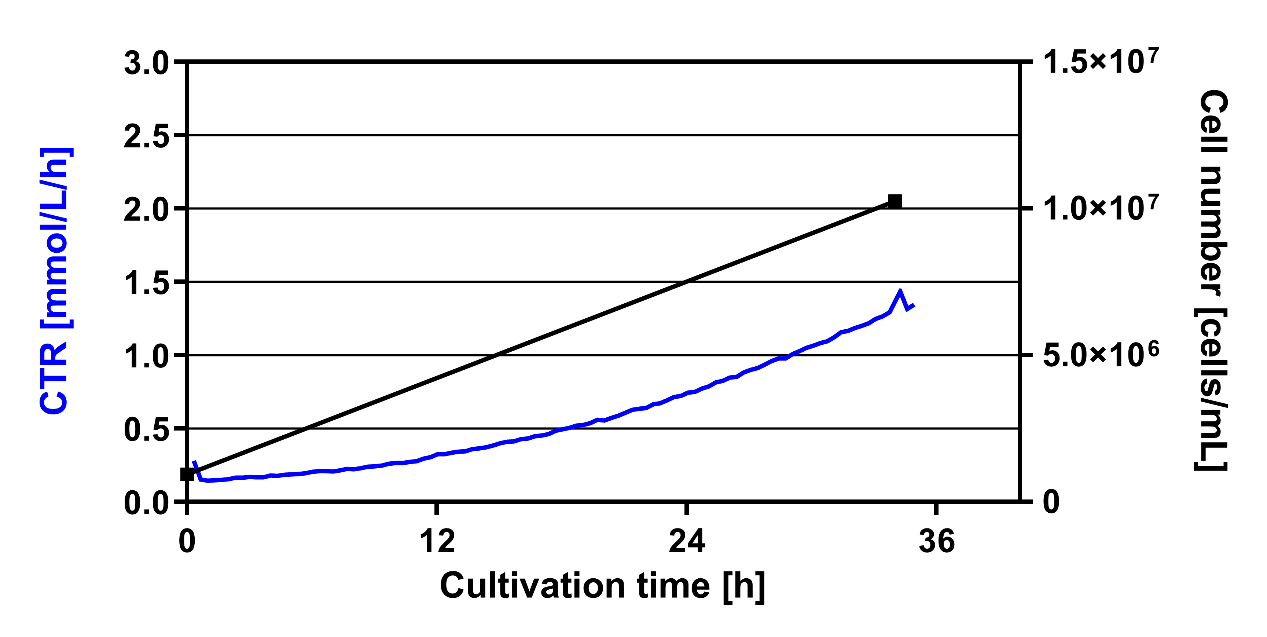


**Supplementary Fig. 2 | Pre-culture set-up of pChR7 strain *via* transfer rate online measurement (TOM).** For the pre-culture, a 1-L TOM flask with a filling volume of 250 mL was inoculated at 1 × 10^6^ cells/mL from a 50 mL liquid culture. After reaching a cell density of 1 × 10^7^ cells/mL, which corresponded to a CTR of 1.3 mmol/L/h, cells were used to inoculate the 1.6-L STR. Culture conditions: HL5 medium with 10 g/L glucose, 20 µg/mL G418; agitation: 140 rpm; temperature: 22°C.
